# Clinical and laboratory indicators predicting coagulase-negative staphylococci as a cause of bloodstream infection among children below five years of age admitted at a tertiary hospital in Dar es Salaam, Tanzania

**DOI:** 10.1080/07853890.2025.2506507

**Published:** 2025-05-16

**Authors:** Petro Juma, Upendo Kibwana, Anjela Charles, Namala Mkopi, Mabula Kasubi, Lilian Nkinda, Salim Masoud, Joel Manyahi, Mtebe Majigo, Agricola Joachim

**Affiliations:** a Department of Microbiology and Immunology, School of Diagnostic Medicine, Muhimbili University of Health and Allied Sciences, Dar es Salaam, Tanzania; b Department of Medical Laboratory Technology, St John’s University of Tanzania, Dodoma, Tanzania; c Department of Paediatrics and Child Health, Muhimbili National Hospital, Dar es Salaam, Tanzania; d Department of Diagnostic and Laboratory Services, Central Laboratory Pathology, Muhimbili National Hospital, Dar es Salaam, Tanzania

**Keywords:** Clinical indicators, laboratory indicators, bloodstream infection, coagulase-negative staphylococci, antimicrobial resistance, under-five children

## Abstract

**Background:**

Coagulase-negative staphylococci (CoNS) are the most commonly isolated bacteria from blood cultures and the most considered contaminants. We conducted a study to assess clinical and laboratory indicators predicting CoNS as a cause of bloodstream infections using two sets of blood cultures among under-five children in Dar es Salaam, Tanzania. In addition, we determined the antimicrobial susceptibility patterns of CoNS.

**Materials and methods:**

This cross-sectional study involved 246 children clinically diagnosed with bloodstream infections admitted to a tertiary hospital . Two sets of blood cultures were collected per patient. Blood samples were tested for microbial growth and antimicrobial susceptibility. Indicators independently predicting CoNS as a cause of bloodstream infection were calculated by binary logistic regression analysis, receiver operating characteristic (ROC) curve analysis to assess the diagnostic performance of predictors. A *p*-value <0.05 at 95% confidence intervals was considered significant.

**Results:**

Of 246 patients, 100(40.7%) were positive blood cultures. CoNS were the most prevalent, isolated in 51(51.0%) blood cultures. Of 51 blood cultures with CoNS, 40(78.4%) were isolated in both blood culture bottles of a set and were regarded as causative of bloodstream infection,of this 34(85.0%) were methicillin resistance CoNS. Multivariate analysis identified tachycardia (aOR = 14.69, 95%CI 1.36–158.42, *p* = 0.027) and *in situ* intravenous cannulation (aOR = 66.75, 95%CI 3.61–1234.40, *p* = 0.005) as significant predictors of CoNS bloodstream infection, with a prediction score of 94.1%. The ROC curve analysis demonstrated tachycardia and *in situ* intravenous cannulation had AUC > 0.7 (*p* < 0.05). The CoNS were frequently resistant to penicillin (97.5%), erythromycin (82.5%), and trimethoprim-sulfamethoxazole (77.5%).

**Conclusions:**

CoNS remains the most common bacteria causing bloodstream infections. *In situ* intravenous cannulation and tachycardia were potential clinical indicators in improving early diagnosis of CoNS as a cause of bloodstream infections and guiding timely treatment decisions. High antimicrobial resistance observed necessitating strengthening of antimicrobial stewardship.

## Introduction

Coagulase-negative staphylococci (CoNS) are commensals of human skin and mucosa membranes [1]. They are the most commonly isolated bacteria from blood cultures and are regarded as the most contaminant [2]. However, CoNS are reported as a common cause of nosocomial infections, such as bloodstream infections (BSI) in children under the age of five years, who frequently use indwelling medical devices as life-saving procedures [3,4]. In Africa, the magnitude of CoNS in blood cultures varies widely among countries, ranging from 6% to 68% [5]. In Tanzania, the magnitude of CoNS in blood cultures differs across various healthcare institutions and age groups, ranging from 35.6% to 67.4% [6,7].

Coagulase-negative staphylococci (CoNS) are increasingly recognized as a cause of bloodstream infections (BSI) in hospitalized children, particularly those under five years of age. These infections are often associated with medical devices such as intravenous cannulas and central venous catheters [8]. Studies from Sub-Saharan Africa (e.g. Nigeria, South Africa) and other regions (e.g. India, Brazil) have reported varying CoNS-BSI prevalence, ranging from 15% to 45% in neonatal and pediatric intensive care units [5,9]. Despite these variations, there is limited data from Tanzania, highlighting the need for region-specific research on risk factors and antimicrobial resistance patterns. Most healthcare facilities collect one set of blood samples for culture versus the recommended two to three sets which improve diagnostic accuracy and distinguish true bloodstream infections from contamination due to the high-cost implications, especially in resource-limited countries like Tanzania [10]. Additionally, global studies suggest that differentiating true CoNS infections from contamination remains a major challenge, particularly in resource-limited settings where standardized diagnostic protocols are not widely implemented [5].

In addition, CoNS isolated from children with BSI in our settings have a wide range of resistance to most antimicrobial agents such as penicillin, erythromycin, gentamycin, clindamycin, and oxacillin [6,7]. High prevalence of methicillin-resistant CoNS (MR-CoNS), which limits treatment options. Resistance in CoNS is primarily mediated by the *mecA* gene, which encodes penicillin-binding protein 2a (PBP2a), conferring resistance to β-lactam antibiotics, including methicillin and oxacillin [11]. The presence of other resistance determinants, such as *erm* (macrolide resistance) and *aac (6′)-Ie-aph (2″)-Ia* (aminoglycoside resistance) genes, further complicates treatment decisions which causes prolonged hospitalizations, increased costs, morbidity, mortality, and unnecessary antibiotic use, thus in­creasing the risk of the emergence of antimicrobial resistance [12]. The increasing emergence of multidrug-resistant (MDR) CoNS underscores the need for comprehensive antimicrobial stewardship programs, particularly in pediatric care settings.

Distinguishing between true CoNS bloodstream infections and contamination remains a major challenge in clinical practice [10]. Many cases of CoNS growth in blood cultures are dismissed as contaminants, leading to potential under-diagnosis of true infections. Molecular techniques such as polymerase chain reaction (PCR)-based detection of species-specific genes such as *icaADBC*, *atlE* and biofilm formation assays have been proposed as additional tools to differentiate true infections from contaminants [13,14]. Additionally, multiplex PCR and matrix-assisted laser desorption/ionization-time of flight (MALDI-TOF) mass spectrometry have shown promise in identifying pathogenic CoNS strains, particularly in resource-rich settings [14,15]. However, these techniques remain underutilized in low-resource settings, necessitating improved diagnostic strategies to enhance clinical decision-making. As a result, various clinical and laboratory criteria have been proposed to assist in differentiating CoNS-BSI from contamination [15,16]. Clinical indicators such as fever, hypothermia, an elevated heart rate, an increased respiratory rate, long-term intravascular catheterization, *in situ* intravenous cannulation, history of invasive procedures, presence of co-morbidities such as HIV/AIDS, cancer, hospitalization for more than 48 h, admission to the intensive care unit (ICU), and specific age groups, such as neonates and infants [16,17]. Moreover, laboratory criteria such as isolation of CoNS in two sets of blood cultures, bacteria culture growth within ≤48 h, increased or decreased total white blood cell count (leukocytosis/leukopenia), increased or decreased neutrophil count (neutrophilia/neutropenia), and patients with increased C-Reactive Protein (CRP) have also been reported [18].

Despite CoNS being multidrug-resistant and more predominantly bacteria isolated from children under five with BSI [6,7], most health facilities have no established guidelines to differentiate CoNS-BSI from contamination. This study assessed the clinical importance of CoNS using two sets of blood cultures, alongside clinical and other laboratory indicators, to predict CoNS-BSI and antimicrobial susceptibility patterns in children below five years of age at Muhimbili National Hospital (MNH), Dar es Salaam, Tanzania. Data obtained from this study will facilitate the development of guidelines to differentiate CoNS-BSI from contamination and update the antibiotic susceptibility profile specific to our setting, aiding in the accurate diagnosis and management of CoNS-BSI in under-five children.

## Materials and methods

### Study design and setting

A hospital-based cross-sectional study was conducted at MNH, Dar es Salaam, Tanzania, from March to July 2023. MNH is a tertiary hospital with a 1,500-bed capacity. It also serves as a university teaching hospital and a national referral hospital. It attends 2,000 outpatients daily and admits up to 1,200 inpatients weekly. Approximately 230 blood cultures are performed monthly for children under five years of age using one set of blood cultures.

### Study population, sample size, and sampling technique

The study involved children aged one month to five years of age admitted at MNH with clinically suspected BSI. Children were recruited from various units within the pediatrics department, including the general pediatric, pediatric intensive care unit, infectious/diarrhea, oncology and pediatric surgery wards. The study included children aged one month to five years presented with symptoms and clinical signs of bloodstream infection such as hyperthermia (≥ 38 °C) or hypothermia (< 36 °C), age specific tachypnea and tachycardia, altered state of consciousness, convulsion and inability to feed. Children who could not collect two sets of blood samples were excluded from the study. The minimum sample size estimated was 246 children aged one month to five years using standard statistical power calculations, aiming for an 80% power, to detect a minimum difference of 6.2% in CoNS-positive cases, assuming a prevalence of 20% from previous reported proportion of CoNS in BSI among under-five children [19]. A convenient sampling technique was used to recruit children from five different wards across the pediatric department. However, the total number of participants recruited from each ward was based on probability proportional to size sampling.

### Data and sample collection

We used structured questionnaires to collect data, including demographics and clinical characteristics from parents/guardians. Trained phlebotomists aseptically collected two sets of blood cultures into BD BACTECTM Peds Plus/F Culture vials (Becton Dickinson Company, USA). A blood sample was collected from different venipuncture sites, either simultaneously or at intervals of 30 min to one hour, depending on the patient’s clinical condition, in order to increase the likelihood of detecting pathogens causing bloodstream infections. The volume of blood collected per culture was based on the patient’s age [20]. Laboratory findings such as the isolation of CoNS from two sets of blood cultures, the time to culture positivity, antimicrobial susceptibility patterns of CoNS isolated from two sets, total WBC count, Neutrophil count, and CRP were collected from the central pathology laboratory during sample processing.

### Isolation and identification

The inoculated blood culture vials were incubated aerobically at 35–37 °C in the BD BACTEC^TM^ FX200 blood culture system (Becton Dickinson Company, USA) for a maximum of 5 days. Flagged positive blood cultures were sub-cultured on Columbia Blood agar supplemented with 5% sheep blood (Oxoid Ltd, Chester, UK) and MacConkey agar with crystal violet (Oxoid Ltd., Chester, UK). Inoculated plates were incubated at 35 ± 2 °C for 18–24 h, and 5% Carbon dioxide (CO_2_) for blood agar plates only. Bacterial/yeast isolates were identified by colonial morphology on culture media, Gram stain, and biochemical tests including catalase test, free and bound coagulase test, and mannitol salt agar (Oxoid Ltd., Chester, UK) for gram positive bacteria. For gram negative bacteria oxidase, Analytical Profile Index (API) 20E and API 20-NE (bioMérieux SA, Lyon, France) were performed and germ tube was performed for gram positive yeast cells. The CoNS isolated from two sets of blood cultures with identical antibiotic susceptability were considered as a causative of BSI while isolation of CoNS from a single set of blood culture were regarded as contaminants [10].

### Antimicrobial susceptibility testing

Antimicrobial susceptibility testing was performed using the Kirby-Bauer disk diffusion method according to the Clinical Laboratory Standard Institute, 2022 guideline [21]. Using a sterile cotton swab, we prepared the inoculum by touching four distinct pure colonies and emulsifying them in 3 ml of sterile physiological saline. We standardized a suspension with 0.5 McFarland standard using a McFarland Densitometer. Lawn culture was performed on the Mueller-Hinton agar (Oxoid Ltd., Chester, UK) plates and incubated at 35 °C for 18–24 h. The antimicrobials used included; gentamycin (10 µg), erythromycin (15 µg), penicillin (10 units), ciprofloxacin (5 µg), trimethoprim/sulfamethoxazole (1.25/23.75 µg), clindamycin (2 µg), and chloramphenicol (30 µg), and cefoxitin (30 µg). Cefoxitin disk diffusion tests were used to identify methicillin-resistance CoNS (MR-CoNS).

### Quality control

All culture media were prepared according to the manufacturer’s instructions. *Staphylococcus aureus* ATCC 25923 was used as the standard control organism for a performance check of the prepared culture media and antibiotic disks. A sterility check was done by randomly selecting 10% of the prepared batch of the culture media, incubating them at 35–37 °C for 18–24 h, and observing the presence or absence of any growth. Negative and positive controls were included to validate the performance of biochemical tests and testing reagents.

### Data analysis

Data analysis was performed using Statistical Package for Social Science (SPSS) version 20.0 (IBM SPSS, Chicago. IL, USA). Categorical variables were presented in proportions, while continuous variables were presented using a median with an interquartile range (IQR). The chi-squared test (Pearson’s or Fisher’s exact test) was used to compare the baseline characteristics for the categorical and continuous variables associated with CoNS-BSI. Multicollinearity was assessed to determine if independent variables were highly correlated using variance inflation factor (VIF), to ensure stability and reliability of regression coefficients. Variables with VIF >10, 5–10, <5 were considered as highly collinearity, moderate collinearity, low collinearity respectively. Variables that showed a significant association with CoNS-BSI in the univariate analysis (*p* < 0.05) and have low collinearity were included in the multivariate analysis. Multivariate logistic regression was used to identify independent predictors of CoNS-BSI. To further assess the diagnostic performance of identified predictors, receiver operating characteristic (ROC) curve analysis was performed, calculating the area under the curve (AUC) to determine the sensitivity and specificity. A *p*-value <0.05 at 95% confidence intervals was considered statistically significant. The predictive score for CoNS-BSI was calculated by creating composite variables.

## Results

### Demographic and clinical characteristics of the study participants

We enrolled 246 children below five years of age with clinical suspicion of BSIs. Half of the participants, 125 (50.8%), were aged 1–12 months. The median age of participants was 12 months, interquartile range (IQR); 7 to 36 months. More than half, 138 (56.1%), were male, and the majority, 145 (58.9%), were recruited from the general pediatric wards. Most 220 (89.4%), had up to five days of hospital stay, with a median of 3 days (IQR: 2 to 4 days). Furthermore, 197 (80.1%) had in-situ intravenous cannulation, while 100 (40.7%) had heart rates greater than 170 beats per minute (bpm). Seventy-three participants, (29.7%), had respiratory rates greater than 40 breaths per minute (bpm), with a median respiratory rate of 35 bpm (IQR: 28 to 42 bpm). A small proportion of participants, 22 (8.9%), had other co-morbidities. The vast majority of the study participants, 229 (93.1%), had received antibiotic treatment before enrollment (Table 1).

**Table 1.** Demographic and clinical characteristics of the study participants *N* = 246.

	Variable	Frequency (n)	Percent (%)
Age group (months)	1–12	125	50.8
	13–24	36	14.6
	25–36	52	21.1
	37–60	33	13.4
	Median age in months (IQR)	12 (7, 36)	
Sex	Male	138	56.1
	Female	108	43.9
Site of admission	General pediatric unit	145	58.9
	Pediatric ICU	57	23.2
	Infectious unit	19	7.7
	Oncology unit	14	4.7
	Pediatric surgical ward	11	4.5
Risk factors for BSI	CVC	53	21.5
	*In situ* IV cannulation	197	80.1
	Intubation	35	14.2
	Surgery	9	3.7
Heart rate (beats/min)	≤ 100	1	0.4
	101–170	145	58.9
	> 170	100	40.7
	Median heart rate (bpm) (IQR)	141.5 (125.0, 178.0)	
Respiratory rate (breaths/min)	≤ 30	82	33.3
	31–40	91	37.0
	> 40	73	29.7
	Median respiratory rate (bpm) (IQR)	35.0 (28.0, 42.0)	
Duration of hospitalization (days)	≤ 5	220	89.4
	6–11	20	8.1
	> 12	6	2.4
	Median duration of hospitalization (days) (IQR)	3 (2, 4)	
Co-morbidities (*n* = 22)	Cancer	12	54.5
	Chronic kidney disease	5	22.7
	Sickle cell disease	2	9.1
	Other co-morbidities (TB, ITP, CD)	3	13.6
On antibiotic use prior sample collection		229	93.1

IQR-interquartile range, ICU-intensive care unit, CVC-central venous catheter IV: intravenous, TB: tuberculosis, ITP: immune thrombocytopenia CD: coagulation disorder.

### The proportion of bacteria and fungi isolated from blood culture

Of 246 patients, 100 (40.7%) had positive blood culture results. Among these, 74 (74.0%) had growth of the same bacterial colonies/morphology in two sets of blood cultures. Out of the 100 positive blood cultures, the majority, 81 (81.0%), were gram-positive cocci bacteria, with CoNS being the most prevalent, accounting for 51 (51.0%) cases, followed by *S. aureus* 28 (28.0%). Of the 51 sets with CoNS isolated, 40 (78.4%) had two sets of blood cultures with CoNS isolates and were thought to be the cause of CoNS-BSIs, while 11 (21.6%) grew in a single set of blood cultures and were thought to be contaminants. Gram-negative ­bacteria account for 14 (14.0%), while 5 (5.0%) were gram-positive yeast cells (Table 2).

**Table 2.** Proportion of isolated bacteria and fungi from blood culture.

Variable		Frequency (n)	Percent (%)
Culture results	Bacterial growth	100	40.7
	No bacterial growth	146	59.3
Blood culture	Growth in one set	26	26.0
	Growth in two sets	74	74.0
Gram stain reaction	Gram positive cocci	81	81.0
	Gram negative bacteria	14	14.0
	Gram positive yeast cells	5	5.0
Isolated pathogens	CoNS	51	51.0
	*Staphylococcus aureus*	28	28.0
	Other GPC	2	2.0
	*E. coli*	4	4.0
	*Klebsiella pneumoniae*	3	3.0
	*Pseudomonas aeruginosa*	2	2.0
	Other GNR	5	5.0
Number blood culture sets CoNS isolated	Single set	11	21.6
	Two sets	40	78.4

CoNS = coagulase-negative staphylococci, Other GPC = Gram-positive bacteria (*Streptococcus pneumoniae* (*n* = 1) and *Enterococcus species* (*n* = 1)). Other GNR = Gram-negative bacteria (*Enterobacter cloacae* (*n* = 1), *Acinetobacter baumannii* (*n* = 2)*, Citrobacter freundii (n* = 1*)*, and *Serratia marcescens* (*n* = 1)).

### Distribution of CoNS isolates across demographic, clinical, and laboratory indicators of BSI

Children with a heart rate > 170 beats/min, 35 (87.5%), a respiratory rate greater than 40 breaths/min 33(86.8%), and in-situ intravenous cannulation 37 (84.1%) had a significantly higher proportion of CoNS-BSI (*p* < 0.05). No significant variations in the proportion of CoNS-BSI were observed among the participants based on age, sex, location of admission, duration of hospital stay, central venous catheter, intubation, and current surgery (*p* > 0.05). Children with a total WBC count less than 5.0 (K/µL), 23 (88.5%), and a neutrophil count greater than 80.0%, 34 (87.2%) exhibited significant a higher proportion of CoNS-BSI than the counterpart (*p* < 0.05). No significant difference was observed in CRP level and time of positivity (Table 3).

**Table 3.** Distribution of CoNS isolates across demographic, clinical, and laboratory indicators of BSI.

Variable		CoNS-BSI (*n* = 40)	CoNS-contaminants (*n* = 11)	
		n (%)	n (%)	*p*-value
Age (months)	1–12	27 (81.8)	8 (18.2)	0.488
	> 12	13 (72.2)	5 (27.8)	
Sex	Male	23 (88.5)	3 (11.5)	0.076
	Female	17 (68.0)	8 (32.0)	
Heart rate (beats/min)	≤ 170	5 (45.5)	6 (54.5)	**0.007**
	> 170	35 (87.5)	5 (12.5)	
Respiratory rate (breaths/min)	≤ 40	7 (53.8)	6 (46.2)	**0.021**
	> 40	33 (86.8)	5 (13.2)	
Admission unit/ward	General pediatric unit	29 (87.9)	4 (12.1)	0.107
	Pediatric ICU	4 (57.1)	3 (42.9)	
	Infectious unit	5 (71.4)	2 (28.6)	
	Oncology unit	1 (50.0)	1 (50.0)	
	Pediatric surgical ward	1 (50.0)	1 (50.0)	
Duration of current hospitalization (days)	≤ 3	22 (75.9)	7 (24.1)	0.737
	> 3	18 (81.8)	4 (18.2)	
Central venous catheter	Yes	4 (57.1)	3 (42.9)	0.162
	No	36 (81.8)	8 (18.2)	
Intravenous cannulation	Yes	37 (84.1)	7 (15.9)	**0.031**
	No	3 (42.9)	4 (57.1)	
Intubation	Yes	3 (75.0)	1 (25.0)	1.000
	No	37 (78.7)	10 (21.3)	
Current surgery	Yes	1 (50.0)	1 (50.0)	0.388
	No	39 (79.6)	10 (20.4)	
Total WBC count (K/µL)	˂ 5.0	23 (88.5)	3 (11.5)	**0.036**
	5.0–26.0	3 (42.9)	4 (57.1)	
	> 26	14 (77.8)	4 (22.2)	
Neutrophil count (%)	˂ 40.0	3 (42.9)	4 (57.1)	**0.018**
	40.0–80.0	3 (60.0)	2 (40.0)	
	> 80	34 (87.2)	5 (12.8)	
C-reactive protein (mg/L)	≤ 5.0	7 (77.8)	2 (22.2)	1.000
	> 5	33 (78.6)	9 (21.4)	
Time to positivity (hours)	≤ 48	34 (82.8)	7 (17.1)	0.193
	> 48	6 (60.0)	4 (40.0)	

CoNS: coagulase-negative staphylococci, BSI: bloodstream infection, ICU: intensive care unit, WBC: white blood cell.

### Predictors for CoNS bloodstream infections

In the univariate logistic regression analysis, patients with heart rate greater than 170 beats/minute had eight times increased odds of having CoNS-BSI than those with heart rate below 170 beats/minute (Crude Odds Ratio (cOR) = 8.4, 95% confidence interval (CI) 1.85–38.11, *p* = 0.006). Patients with a respiratory rate greater than 40 breaths/minute had five times increased odds of having CoNS-BSI than those with a respiratory rate below 40 breaths/minute (cOR = 4.8, 95%CI 1.16–19.80, *p* = 0.030). The odds of having CoNS-BSI were seven times higher among patients who had *in situ* intravenous cannulation compared to those who did not have (cOR = 7.1, 95%CI 1.30–38.62, *p* = 0.024). Patients with a WBC count of less than 5.0 cells/µL had ten times increased odds of having CoNS-BSI than those with a normal WBC count (cOR = 10.2, 95%CI 1.50–69, *p* = 0.018). In the multivariate logistic regression analysis, heart rate and *in situ* intravenous cannulation were the independent factors that appeared to predict CoNS-BSI. Patients with heart rate greater than 170 beats/minute had 14.7 times higher odds of developing CoNS-BSI (an adjusted odds ratio (aOR) = 14.69, 95%CI 1.36–158.42, *p* = 0.027) compared to patients with heart rate below 170 beats/minute. Those who had *in situ* intravenous cannulation had 66.7 times higher odds of having CoNS-BSI (aOR = 66.75, 95%CI 3.61–1234.40, *p* = 0.005) than those who did not have (Table 4).

**Table 4.** Univariate and multivariate analysis of the factors predicting CoNS-BSI.

Variable	Category	Total N	CoNS-BSIs (%)	Univariate analysis		Multivariate analysis	
				cOR (95% CI)	*p*-value	aOR, 95% CI	*p*-value
Age in months	1–12	33	27 (81.8)	1.73 (0.44–6.74)	0.429		
	> 12	18	13 (72.2)	Ref			
Sex	Male	26	23 (88.5)	3.61 (0.83–15.65)	0.087		
	Female	25	17 (68.0)	Ref			
Heart rate (beats/min)	> 170	40	35 (87.5)	8.4 (1.85–38.11)	**0.006**	14.69 (1.36–158.42)	**0.027**
	≤ 170	11	5 (45.5)	Ref			
Respiratory rate (breaths/min)	> 40	37	32 (86.5)	4.8 (1.16–19.80)	**0.030**	0.40 (0.03–6.30)	0.515
	≤ 40	14	8 (57.1)	Ref			
Central venous catheter	Yes	7	4 (57.1)	0.30 (0.06–1.59)	0.156		
	No	44	36 (81.8)	Ref			
Intravenous cannulation	Yes	44	37 (84.1)	7.05 (1.30–38.62)	**0.024**	66.75 (3.61–1234.40)	**0.005**
	No	7	3 (42.9)	Ref			
Intubation	Yes	4	3 (75.0)	0.81 (0.08–8.66)	0.862		
	No	47	37 (78.7)				
Surgery	Yes	2	1 (50.0)	0.26 (0.01–4.47)	0.351		
	No	49	39 (79.6)	Ref			
Site of admission	General PU	33	29 (87.9)	7.25 (0.37–140.20)	0.190		
	PICU	7	4 (57.1)	1.33 (0.06–31.10)	0.858		
	Infectious unit	7	5 (71.4)	2.50 (0.10–62.60)	0.577		
	Oncology unit	2	1 (50.0)	1.00 (0.02–50.40)	1.000		
	Pediatric SW	2	1 (50.0)	Ref			
Total WBC count (K/µL)	˂ 5.0	26	23 (88.5)	10.22 (1.50–69.76)	**0.018**	24.31 (0.70–838.81)	0.077
	5.0–26.0	7	3 (42.9)	Ref			
	> 26.0	18	14 (77.8%)	4.67 (0.72–30.11)	0.105		
Neutrophil count (%)	˂ 40.0	7	3 (42.9)	0.5 (0.05–5.15)	0.560		
	40.0–80.0	5	3 (60.0)	Ref			
	> 80.0	39	34 (87.2)	4.53 (0.60–34.19)	0.143		
C-reactive protein value (mg/L)	>5.0	42	33 (78.6)	1.05 (0.185–5.94)	0.958		
	≤ 5.0	9	7 (77.8)	Ref			/

cOR: crude odds ratio, aOR: adjusted odds ratio, Ref: reference category, PU: pediatric unit, PICU: pediatric intensive care unit, SW: surgical ward, WBC: white blood cell.

### Predictive score for CoNS bloodstream infections

**Figure 1.**
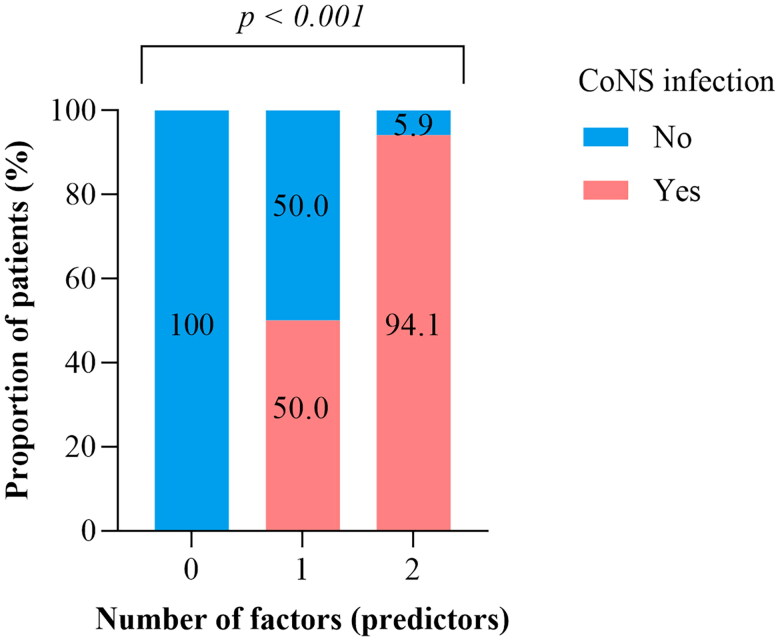
Predictive score for tachycardia and *in situ* intravenous cannulation as indicators predicting CoNS-BSI.

It was found that a heart rate greater than 170 beats/min and *in situ* intravenous cannulation had a higher likelihood of predicting CoNS-BSI, *p*-value ˂0.001. Without any predictor, the possibility of patients having CoNS-BSI was 0.0%. In the presence of one predictor, the possibility increased to 50.0%, and with two predictors present, the possibility increased to 94.1%. Children with heart rate greater than 170 beats/min and *in situ* intravenous cannulation had 94.1% of predicting CoNS-BSI (Figure 1).

### Diagnosis performance of indicators predicting CoNS-BSI

**Figure 2.**
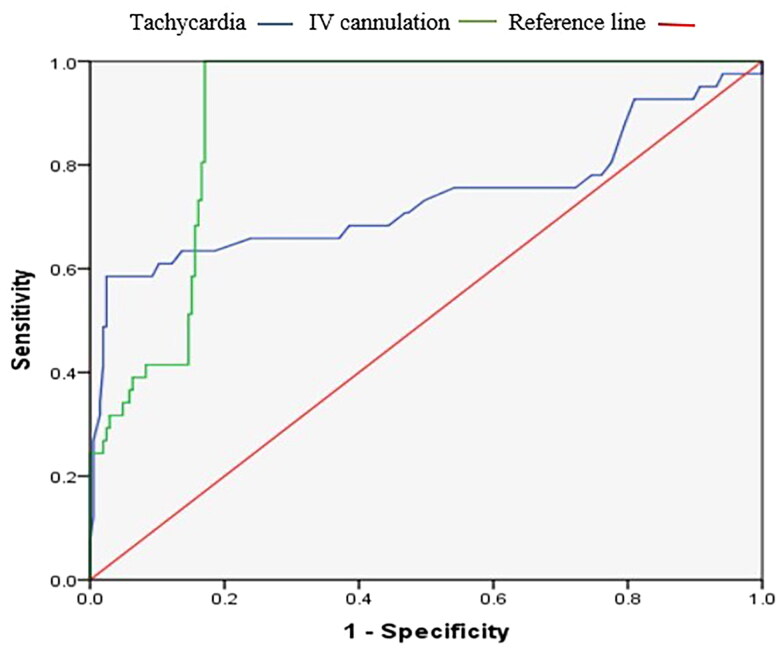
The ROC curves analysis applied to test performance characteristics of tachycardia and *in situ* intravenous cannulation in predicting CoNS-BSI.

The area under the curve (AUC) for tachycardia, and *in situ* cannulation to predict CoNS-BSI were 0.735 (95% CI: 0.626–0.844, Sensitivity 75.6%, and Specificity 45.9%), and 0.898 (95% CI: 0.859–0.937, Sensitivity 92.7%, Specificity 82.9%), respectively, all with *p* < 0.05 (Table 5; Figure 2).

**Table 5.** Performance characteristics of tachycardia and *in situ* intravenous cannulation in predicting CoNS-BSI.

Indicators	AUC	95% CI	P value	Cut-off	Sensitivity (%)	Specificity (%)
Tachycardia	0.735	0.626–0.844	0.000	128.5	75.6	45.9
IV cannulation	0.898	0.859–0.937	0.000	0.874	92.7	82.9

AUC: Area under the curve, CI: Confident interval.

### The antimicrobial resistance patterns of CoNS

**Figure 3.**
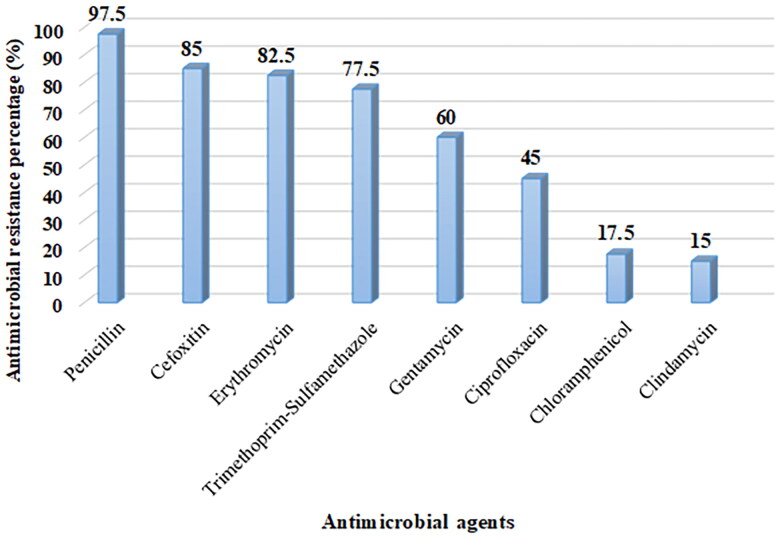
Antimicrobial resistance patterns of CoNS causing BSI.

The CoNS exhibited high levels of resistance to several antibiotics. The majority of the isolates were highly resistant to penicillin (97.5%), cefoxitin (85.0%) indicating MR-CoNS, and erythromycin (82.5%). Of 34 CoNS susceptible to clindamycin, 4 (11.8%) exhibited inducible clindamycin resistance (Figure 3).

## Discussion

This study assessed the clinical importance of CoNS using two sets of blood cultures, alongside clinical and other laboratory indicators, to predict CoNS-BSI among children below five years. CoNS-BSI were more prevalent and accounted for (40%) of the bacteria isolated. Other studies have also reported similar trends [7,22]. In contrast to the findings, a low proportion of CoNS-BSI has also been reported [23]. A high proportion of CoNS-BSI from our study might be due to using two sets of blood cultures from different sites, which increases the positivity rate and classifies CoNS-BSI versus contaminant CoNS compared to using a single blood culture set [24,25].

In the current study, the sole clinical indicators predicted CoNS-BSI were tachycardia (heart rate >170 beats per minute) and *in situ* intravenous cannulation. Tachycardia occurs in patients with infections due to the regulation of host defense, including the production of pro-inflammatory cytokines and physiological responses that cause changes in heart rate and heart rate variability [26]. Nevertheless, indwelling of medical devices like IV cannula favors CoNS to gain access into the bloodstream, which in turn causes infection [3]. In the presence of two predictors, the possibility for CoNS-BSI increased to more than 90.0%. Our findings indicate that the development of a predictive score can be used to estimate the patients likely to be infected with CoNS, and this proportion increases as the number of predictors increases [18,27]. In addition, both indicators had more than 75% sensitivity in predicting CoNS-BSI. Given these findings, clinicians should consider prioritizing blood cultures for patients presenting with unexplained tachycardia, particularly those with prolonged intravenous cannulation. However, these markers should be interpreted alongside labo­ratory parameters such as WBC count and CRP to improve diagnostic accuracy and minimize unnecessary antibiotic use [28].

Regarding laboratory indicators, this study found that a low WBC count increased the likelihood of CoNS-BSI. This finding partially corresponds with those reported in the previous studies that linked CoNS-BSI with deviations in WBC levels, specifically leukopenia, WBC count <4,000 cells/µL or >12,000 cells/µL, and total WBC, <1000 cells/µL or > [19, 31] respectively. Furthermore, a trend was noticed toward BSI in cases where the total WBC count was below 5.0 K/µL, indicating that this predictor has specificity [29,30]. The similarity observed could be due to the immunological response of the study participants against CoNS infections, which might trigger an immune response that affects WBC levels, both excessively low (leukopenia) and high (leukocytosis), also could be due to the severity of infection [31,32].

No significant associations were found between an elevated total WBC count (>26 K/µL), neutrophil count, CRP levels, and time to positivity with CoNS-BSI. These findings differ from other studies, which suggested that higher total WBC count/neutrophil counts, time to positivity, and elevated CRP were indicative of CoNS-BSI [27,28]. Moreover, these findings also contradict the conclusions drawn in other studies [15,19]. The observed difference could be due to the stage of infection at which the measurements were taken, variations in immune responses, and underlying conditions that might contribute to varying immune reactions and subsequent associations with BSI [33]. In the current study, a small sample was used, which might have affected the power of the study, leading to a lack of association. The current study identified tachycardia and *in situ* intravenous cannulation as significant predictors of CoNS bloodstream infection. However, the integration of laboratory markers such as elevated WBC count, increased neutrophil percentage, and high CRP levels could further enhance the predictive value of these clinical signs. Previous studies have shown that patients with CoNS-BSI often present with WBC counts >12,000/mm³ and CRP levels >10 mg/L, suggesting a heightened inflammatory response [28]. Future research should explore combining clinical and laboratory markers to develop a more robust predictive model for CoNS-BSI.

Furthermore, a high resistance rate was observed in commonly used antibiotics such as penicillin, erythromycin, trimethoprim-sulfamethoxazole, and ciprofloxacin; other studies reported similar trends [6,7]. In keeping with other studies, about 86% of pathogenic CoNS were identified as MR-CoNS [6,34,35]. Our finding implies that the majority of CoNS causing BSI in children are multidrug-resistant, this highlights the need for antimicrobial stewardship. Multidrug-resistant CoNS, particularly strains resistant to methicillin, aminoglycosides, and macrolides, which causes difficult-to-treat infection and significantly increases prolonged hospitalizations and unnecessary antibiotic use, which facilitates the emergence of antimicrobial resistance, morbidity, and mortality [12]. Studies from Sub-Saharan Africa have reported that multidrug-resistant CoNS-BSI in pediatric patients lead to longer ICU stays (average 5–7 days additional hospitalization) and increased mortality, particularly in neonates [35]. Given these findings, there is an urgent need for antimicrobial stewardship programs that focus on restricting the overuse of broad-spectrum antibiotics and promoting routine susceptibility testing to guide treatment decisions.

Furthermore, the high prevalence of methicillin-resistant MR-CoNS highlights the need for antimicrobial stewardship. Given the increasing resistance to first-line β-lactam antibiotics, empirical therapy for suspected CoNS-BSI should consider vancomycin or linezolid until susceptibility results are available, although this need to be investigated. Additionally, local antibiograms should be updated regularly to inform empiric treatment guidelines and minimize inappropriate antibiotic use [36].

Our study showed the clinical importance of using two sets of blood cultures to differentiate CoNS-BSI from contaminants. Potential confounders such as age and co-morbidities, were adjusted for through multivariate regression to minimize their influence on our findings.

### Study limitation

We acknowledge the following limitations in our study i) Unable identify CoNS isolates to the species level ii) Molecular typing of CoNS causing BSI was not performed, which could provide more details on virulence genes aiding in identification of CoNS responsible for BSI, and the detection of antibiotic resistance genes that explain mechanisms of resistance. iii) A small sample size limits the number predictors of CoNS-BSI. iv) The study was conducted from March to July 2023 hence, the timeframe may not fully account for potential seasonal variations in bloodstream infections, including CoNS prevalence. In light of these findings, we recommend further studies with a year-round data collection approach involving a larger sample size to determine further the role of clinical and laboratory indicators in predicting CoNS-BSI. In addition, two blood culture sets should be implemented to determine the clinical significance of CoNS for proper patient management in our settings.

## Conclusion

Coagulase-negative staphylococci were the common pathogens causing BSI. *In situ* intravenous cannulation and tachycardia were the independent predictors of CoNS-BSI in children below five years, emphasizing the importance of early identification. Based on these findings, we recommend that hospitalized children presenting with tachycardia (> 170 beats/min) and *in situ* intravenous cannulation should undergo two-set blood cultures before initiating empirical antibiotics. Clinicians should consider CoNS-BSI in differential diagnoses, particularly when these risk factors are present, and antimicrobial therapy should be tailored based on culture and susceptibility results. Furthermore, hospitals should implement infection control protocols that include daily assessment of intravenous catheter necessity, adherence to aseptic insertion techniques, and routine monitoring for signs of catheter-related infections. Intravenous catheters should be promptly removed when no longer clinically indicated, and care bundles promoting proper hand hygiene and catheter maintenance should be reinforced among healthcare workers. Most CoNS causing BSI exhibited a high resistance rate to commonly prescribed antimicrobial agents, underscores the importance of antimicrobial stewardship programs. To address this challenge, national health authorities should prioritize the establishment of antimicrobial resistance surveillance systems specifically monitoring CoNS resistance patterns in pediatric populations. Hospitals should implement routine reporting of MR-CoNS prevalence and treatment outcomes, integrating these data into national antibiograms to guide empirical therapy recommendations. Additionally, antibiotic prescribing guidelines should be regularly updated based on local resistance patterns, and antibiotic stewardship training programs should be expanded to frontline healthcare providers.

## Supplementary Material

CoNS BSI Response to Reviewers comment Final.doc

## Data Availability

The corresponding author can provide the datasets utilized and/or analyzed in the current study upon reasonable request.
