# Supplementary material for: Clinical and laboratory indicators predicting coagulase-negative staphylococci as a cause of bloodstream infection among children below five years of age admitted at a tertiary hospital in Dar es Salaam, Tanzania
Source: Ann Med. 2025 May 16;57(1):2506507. doi: 10.1080/07853890.2025.2506507 (PMC12086907; doi:10.1080/07853890.2025.2506507)
Supplement: CoNS BSI Response to Reviewers comment Final.doc [file IANN_A_2506507_SM4887.doc]

**RESPONSE TO REVIEWER ONE COMMENTS**

| **Reviewers’ comment** | **Authors response** |
| --- | --- |
| **MATERIALS & METHODS**  Clearly describe how convenience sampling was handled to mitigate selection bias | We thank the reviewer for comment given.  A convenient sampling technique was used to recruit children from five different wards across the paediatric department. The total number of participants recruited from each ward was based on probability proportional to size sampling to ensure representativeness from each ward. We have revised the text to include this information (Page 10). |
| The inclusion and exclusion criteria need elaboration (e.g., were neonates or children with underlying comorbidities excluded?). | Thank of the comments. We have clearly elaborated the inclusion and exclusion criteria (Page 9). |
| Specify if the CoNS species were identified and whether molecular typing was performed to distinguish pathogenic from contaminant strains. | Identification of CoNS to species level and molecular typing was not performed in the current study due to financial constraints. We acknowledged this limitation in the Discussion section (Page 22) |
| Clarify the reference standards used for defining true bloodstream infections and how contamination was assessed in repeat cultures. | We appreciated the comment given. True CoNS-BSI in the current study was defined based on identification of the same bacteria isolate from both sets of blood while isolation of CoNS from a single set of blood culture were regarded as contaminants. Antibiotic susceptibility pattern was also used as an additional criterion, (Miller et al.2018), page 10. |
| The criteria for variable inclusion in the multivariate regression model should be explicitly stated. | Thanks for the comment, criteria for variable inclusion in the multivariate regression model has been added. Variables that showed a significant association with CoNS-BSI in the univariate analysis (*p* < 0.05) and have low collinearity were included in the multivariate analysis (Page 11). |
| Address whether multicollinearity was assessed in the logistic regression analysis | Multicollinearity was assessed using variance inflation factor (VIF) in the logistic regression analysis, this has been added in manuscript as suggested (Page 11). |
| Provide confidence intervals and explain how missing data (if any) were handled | Thanks for good comment, confidence intervals were provided (Page 16-Table 4), (Page 18, Table 5).  We had no any missing data in our current study. |
| **RESULTS**  The tables and figures should be better integrated into the narrative, with explicit cross-references in the results section | We thank you for the observation. All the tables and figures have been integrated into the narrative, with explicit cross-references in the results section as suggested. |
| Add clear explanations for the data labels and statistical annotations in the figure legends | Clearly explanation in Figure legend has added (Page 17). |
| Include a visual depiction of the predictive score’s performance (e.g., a ROC curve showing sensitivity, specificity, and AUC). | We thank reviewer for the comment. The ROC curve showing performance characteristics of predators of CoNS-BSI has been added as suggested (Page 18). |
| **DISCUSSION**  Highlight potential confounders that may have influenced the findings | Potential confounders that may have influenced the findings such as age and Co-morbidities which were adjusted through multivariate regression (Page 22), have been added as suggested. |
| Expand on the clinical implications of implementing the predictive score in practice, particularly in terms of antimicrobial stewardship | We appreciate for the given comments, clinical implications of implementing the predictive score in practice, particularly in terms of antimicrobial stewardship has been added as follow;  ‘Given these findings, clinicians should consider prioritizing blood cultures for patients presenting with unexplained tachycardia, particularly those with prolonged intravenous cannulation. However, these markers should be interpreted alongside laboratory parameters such as WBC count and CRP to improve diagnostic accuracy and minimize unnecessary antibiotic use’ (Page 20) |
| **LANGUAGE AND FLOW**  The manuscript requires language editing to correct grammatical errors and improve readability (e.g., "in-situ intravenous cannulation" → "in situ intravenous cannulation"). | We thank reviewer for the observation to improve our manuscript. Grammatical errors have been corrected throughout the manuscript including use of term “in situ intravenous cannulation" as suggested. |
| Page 5, Line 20: Specify the blood volume drawn per culture and the rationale for this range | Thanks for the comment. We used facility SOP for blood culture where blood volume per culture depends on patient’s age. We have revised the text and add this information. ‘A blood sample was collected from different venipuncture sites, either simultaneously or at intervals of 30 minutes to one hour, depending on the patient's clinical condition, in order to increase the likelihood of detecting pathogens causing bloodstream infections. The volume of blood collected per culture was based on the patient's age’ (Huber S et al., Clin Microbiol Infect. 2020 Feb;26(2):168-173. doi: 10.1016/j.cmi.2019.10.006. (Page 10). |
| Table 3: Add a footnote explaining abbreviations (e.g., “BSI”). | List of abbreviation has been added (Page 23) |
| Page 17, Line 40: Include a reference for the clinical significance of heart rates exceeding 170 bpm as a sepsis indicator | We have included a reference for clinical significance of heart rates exceeding 170 bpm as a sepsis indicator in Discussion section as suggested (Huber et al., 2020, Fairchild et al. 2011 (Page 20). |

**RESPONSE TO REVIEWER TWO COMMENTS**

| **Reviewers’ comment** | **Authors response** |
| --- | --- |
| **ABSTRACT**  **Methods and Material**  Justify the use of two blood culture sets. Where to insert: In the Methods section of the abstract, after describing the study design | The justification for use of two blood culture set in the study has been included as suggested (Page 6). |
| **Results**  Emphasize the clinical importance of tachycardia and IV cannulation as predictors. Where to insert: In the Results section of the abstract, when discussing risk factors | The clinical importance of tachycardia and IV cannulation as predator of CoNS-BSI has been included as suggested (Page 6). |
| **Conclusion and Recommendation**  Highlight the clinical implications of antimicrobial resistance. Where to insert: In the Conclusion section of the abstract, after summarizing key findings. | The clinical implications of antimicrobial resistance have been incorporated in the manuscript as suggested (page 6). |
| **INTRODUCTION**  It effectively to outlines the challenge of differentiating CoNS infections from contamination in clinical practice, | We thank reviewer for the comments. We have included information on the challenge of differentiating CoNS infections from contamination in clinical practice particularly in developing countries such as Tanzania as suggested (Page 7) |
| Briefly mention molecular techniques for distinguishing true CoNS infections | We have included the Molecular techniques for distinguishing true CoNS infections including specific genes to be detected has been added as suggested (Page 8). |
| Discuss the role of antimicrobial resistance genes in CoNS infections | The role of antimicrobial resistance genes such as *mecA,* *aac (6′)-Ie-aph (2″)-Ia, erm* in CoNS infections has been added (Page 8). |
| **MATERIAL AND METHODS**  Strengthen the justification for sample size calculation | Sample size calculation justification has been added (Page 9). |
| Acknowledge seasonal variations as a potential limitation | Limitation of seasonal variation of CoNS-BSI prevalence has been included in the manuscript (Page 22) |
| Consider adding an ROC curve or sensitivity analysis for predictive markers | The ROC curve showing performance characteristics of predators of CoNS-BSI has been added as suggested (Page 18). |
| **RESULTS**  Compare findings to previous studies on CoNS-BSI and antimicrobial resistance trends | We appreciate for this constitutive comment. The comparison of CoNS-BSI and antimicrobial resistance trends from previously studies was included in Discussion section page 19, 21. |
| **DISCUSSION**   Integrate laboratory markers (WBC, Neutrophil %, CRP) into the Discussion of predictors | We have added information on the role of integrate laboratory markers such as WBC, Neutrophil percentage, CRP in predicting CoNS-BSI in discussion section as suggested (Page 20-21). |
| Expand the discussion on how MDR-CoNS affects clinical outcomes and antibiotic stewardship | Explanation on how multi-drug resistance CoNS-BSI affects patient’s clinical outcomes and antibiotic stewardship has been included (Page 21). |
| Discuss clinical management strategies in response to high MR-CoNS prevalence | We have added information on clinical management strategies in response to high MR-CoNS prevalence in the discussion section as suggested (Page 21). |
| Provide guidance for clinicians on interpreting tachycardia and IV cannulation as diagnostic markers. | Information to guide for clinicians on interpreting tachycardia and IV cannulation as diagnostic markers has been included (Page 20). |
| **CONCLUSION**  Provide specific recommendations for clinical guidelines on CoNS diagnosis | Recommendation for clinical guidelines on CoNS-BSI diagnosis has been added as advised (Page 22). |
| Suggest hospital infection control strategies to reduce IV cannulation-associated infections | We have added information on how hospital infection control strategies can be employed to reduce IV cannulation-associated (Page 22). |
| Discuss policy recommendations for antimicrobial resistance surveillance in Tanzania | We have reived the text to include policy recommendations for antimicrobial resistance surveillance in Tanzania as suggested (Page 23). |
| **FIGURES & TABLES**  Figure 1 has not been referenced in text | - Figure 1 has been referenced in the manuscript text (Page 17). |
